# Age-specific differences in association of glycosylated hemoglobin levels with the prevalence of cardiovascular diseases among nondiabetics: the National Health and Nutrition Examination Survey 2005–2018

**DOI:** 10.1186/s12872-024-03978-w

**Published:** 2024-06-19

**Authors:** Ruihan Fan, Shuna Li, Zihan Xue, Ruida Yang, Jun Lyu, Hairong He

**Affiliations:** 1 Clinical Research Center, The First Affiliated Hospital of Xi’an Jiaotong University, 277 West Yanta Road, Xi’an, Shaanxi 710061 People’s Republic of China; 2 School of Public Health, Xi’an Jiaotong University Health Science Center, Xi’an, Shaanxi China; 3 Department of Clinical Research, The First Affiliated Hospital of Jinan University, Tianhe District, 613 W. Huangpu Avenue, Guangzhou, Guangdong 510632 People’s Republic of China; 4 Guangdong Provincial Key Laboratory of Traditional Chinese Medicine Informatization, Guangzhou, Guangdong China

**Keywords:** Cardiovascular diseases, Glycated hemoglobin, Nondiabetics, National Health and Nutrition Examination Survey

## Abstract

**Background:**

Previous research has supported the presence of an association between high glycated hemoglobin (HbA1c) levels and cardiovascular disease (CVD). The objective of the present study was to determine whether increased HbA1c levels are associated with high CVD prevalence among nondiabetics. Furthermore, we aimed to explore the possible interaction of HbA1c levels and age in regard to CVD.

**Methods:**

This cross-sectional study analyzed data of 28,534 adult participants in the National Health and Nutrition Examination Survey 2005–2018. The association between HbA1c and CVD was assessed using univariate and multivariate logistic regression models. Propensity score matching was used to reduce selection bias. Subgroup analysis and restricted cubic spline (RCS) were used to further characterize the association between HbA1c levels and CVD. We modeled additive interactions to further assess the relationship between HbA1c levels and age.

**Results:**

In the multivariate logistic regression model, a positive association was found between CVD and increased HbA1c levels (highest quartile [Q4] vs. lowest quartile [Q1]: odds ratio [OR] = 1.277, 95% confidence interval [CI] = 1.111–1.469, *P* = 0.001). In the stratified analyses, the adjusted association between HbA1c and CVD was significant for those younger than 55 years (Q4 vs. Q1: OR = 1.437, 95% CI = 1.099–1.880, *P* = 0.008). RCS did not reveal a nonlinear relationship between HbA1c levels and CVD among nondiabetics (*P* for nonlinearity = 0.609). Additionally, a high HbA1c level was favorably connected with old age on CVD, with a synergistic impact.

**Conclusions:**

Increased HbA1c levels were associated with high CVD prevalence among nondiabetics. However, we still need to carefully explain the effect of age on the relationship between HbA1c and CVD in nondiabetic population. Given the correlations of HbA1c with CVDs and CV events, HbA1c might be a useful indicator for predicting CVDs and CV events in the nondiabetic population.

**Supplementary Information:**

The online version contains supplementary material available at 10.1186/s12872-024-03978-w.

## Introduction

The prevalence of cardiovascular disease (CVD) is becoming more widely acknowledged as a global health concern and is currently the leading cause of mortality [1]. Better management techniques are required for CVDs due to their persistently high incidence rate, which indicates that there is a serious lack of knowledge about the risk factors for cardiovascular (CV) health [1]. Numerous recent investigations have found that even intensive blood glucose control cannot reduce the incidence rate of CV events in patients with diabetes [2]. Studies have found that pathological CV changes in patients with diabetes can occur during the early stages of the associated diseases, and that these changes gradually aggravate with time, and as diseases progress. Intensive blood glucose control cannot reverse the changes that have occurred at the time of disease development. Early detection and intervention to prevent and delay the progression and deterioration of the diseases are effective methods to reduce the frequency of CV events in people with diabetes [3, 4]. The search for biomarkers that predict CVDs or events in nondiabetic populations has therefore become increasingly more important.

Glycated hemoglobin (HbA1c) is currently recognized as an option for the monitoring and chronic treatment of diabetes since it provides a reliable indicator of chronic glycemia and is strongly correlated with the risk of long-term diabetes effects [1]. The analysis of HbA1c in blood provides information about the usual blood glucose levels of an individual during the previous 2–3 months, since this is the expected half-life of red blood cells [2]. HbA1c has been considered as a potential candidate by researchers in the search for new clinical markers that can predict CVD risk. In reality, scientists have been investigating how HbA1c affects both people with and without diabetes in terms of various health consequences. Consequently, increasing amounts of epidemiological data have connected higher HbA1c levels with detrimental health consequences, including cancer, kidney failure, blindness, and CVD, all of which may cause early mortality [3–6]. However, whether increased HbA1c levels are related to the high CVD incidence rate among nondiabetics requires further investigation.

Our study therefore aimed to determine whether the high CVD prevalence among nondiabetics is associated with elevated HbA1c levels using information from the National Health and Nutrition Examination Survey (NHANES) conducted during 2005–2018. Furthermore, we intended to investigate the probable interact relationship of HbA1c levels and age on CVD.

## Materials and methods

### Study population

The NHANES involves the entire USA population and offers a wealth of data regarding the health and diet of the general public. It is worth noting that NHANES uses a complex, multistage probability sampling design and includes oversampling for certain subgroups [7–9]. Comprehensive descriptions of the procedures of the survey can be found on the NHANES website (https://www.cdc.gov/nchs/nhanes/index.htm).

We used data from 2005 to 2018 in this study because it had the complete exposure, outcome, and covariates data we needed. All nonpregnant adult participants (older than 20 years) who underwent the Mobile Examination Center (MEC) from the NHANES during 2005–2018 (*n* = 36,870) were included in our study. Participants were excluded if they had missing or incomplete data on education level, body mass index (BMI), smoking status, sleep disorders, and diabetes records (*n* = 694), or HbA1c values and any CVDs (*n* = 2,061). After excluding participants with diabetes (*n* = 5,581), we had 28,534 people in our final analysis (Supplementary Fig. 1).

### Measurement of HbA1c levels

During the MEC examination, blood was drawn, centrifuged, and kept at < − 20 °C before being analyzed. HbA1c levels were measured at the Diabetes Diagnostic Laboratory University of Missouri School of Medicine using the principles of boronate-affinity and high-performance liquid chromatography. An elaborate description of the HbA1c measurement procedure is available at https://wwwn.cdc.gov/nchs/data/nhanes/2017-2018/labmethods/GHB-J-Premier-508.pdf.

### CVD outcomes

CVD was defined using a combination of the following five self-reported CVD outcomes: congestive heart failure, coronary heart disease (CHD), angina pectoris, heart attack, and stroke [10]. Participants were recorded as having CVDs if they answered “yes” to the following question: “Has a doctor or other health professional ever told you that you had congestive heart failure/coronary heart diseases/angina pectoris/a heart attack/a stroke?” The answers to these questions were collected during individual interviews using a standardized medical-status questionnaire.

### Covariates

For all included participants, demographic variables for age, sex, race (non-Hispanic white, non-Hispanic black, Mexican American, other Hispanic, and other races), and education level (high school or below, college participation, and college graduate or above) were collected during the home interviews. Self-reported medical conditions were used, including sleep disorders (ever informed doctor of trouble with sleeping) and smoking status (having smoked at least 100 cigarettes in their lifetime or not). Standard protocols were followed to assess height, weight, and blood pressure during the MEC exam. The indicators of hypertension were a hypertension diagnosis by a doctor, average systolic and diastolic blood pressures of > 140 and/or 90 mmHg, respectively, or the use of hypertension medication. BMI was categorized into undernourished (< 18 kg/m^2^), normal (18–25 kg/m^2^), overweight (25–30 kg/m^2^), and obese (> 30 kg/m^2^). If the HbA1c level of a participant exceeded 6.5% or they reported receiving a diagnosis from a doctor, they were deemed to have diabetes. Regarding covariates, age, sex, race, education level, BMI, hypertension, sleep disorders, and smoking status were used as categorical variables.

### Statistical analyses

Categorical variables are expressed as numbers and percentages. The baseline characteristics of the various groups were compared using the chi-squared test. Propensity score matching (PSM) was used to reduce bias. The nearest neighbor method was used for matching, using a 1:1 ratio and a caliper distance of 0.2 [11]. HbA1c levels were stratified into four quartiles from lowest (Q1) to highest (Q4). The association between HbA1c levels and CVD was analyzed using univariate and multivariate logistic regression models before and after matching. No factor was adjusted in model (1) Demographic variables including age, sex, education level, and race were adjusted for in model (2) Traditional CV risk variables (smoking status, hypertension, BMI, and sleep disorders) were further adjusted for in model (3) We stratified the aforementioned analyses based on factors that could modify the effects, such as age, sex, and smoking status. Odds ratios (ORs) and 95% confidence intervals (CIs) were also used to summarize the regression analysis results. To further illustrate the correlation between HbA1c levels and CVD, we employed a restricted cubic spline (RCS) with four knots to adapt the model to include the underlying relationship [12].

Finally, an additive interaction model with three indicators was used to investigate the relationship of HbA1c levels, age, and their interactions with CVD prevalence. The model included the relative excess risk of interaction (RERI), attributable proportion of interaction (API), and synergy index (SI) [13]. When 0 was contained in the 95% confidence interval (CI) of RERI and API, or 1 was involved in the 95% CI of SI, there was no interaction between the two variables [14]. Differences with *P* < 0.05 were considered significant. All statistical analyses were performed using SPSS (version 24.0, SPSS, Chicago, IL, USA) and R (version 4.2.2, R Foundation for Statistical Computing, Vienna, Austria).

## Results

### Population characteristics

This cross-sectional study eventually included 28,534 participants. There were 14,682 (51.5%) females, 9,811 (34.4%) with obesity, 12,595 (44.1%) who smoked, and 10,367 (36.3%) with hypertension (Table 1). The prevalence of CVD was 7.9%. Congestive heart failure, CHDs, angina pectoris, heart attack, and stroke were present in 2.0%, 2.9%, 1.7%, 3.0%, and 2.8% of the participants, respectively. Age, sex, education level, race, hypertension, smoking status, BMI, and sleep disorders were all significantly different between the CVD and non-CVD groups (all *P* < 0.001).

**Table 1:** Characteristics of the study population before matching

Characteristic	Overall (*n* = 28,534)	Non-CVD (*n* = 26,269, 92.1%)	CVD (*n* = 2,265, 7.9%)	*P* value
Age (years)				< 0.001
<55years	18,171 (63.7%)	17,687 (67.3%)	484 (21.4%)	
55–<65years	4,517 (15.8%)	4,081 (15.5%)	436 (19.2%)	
≥ 65years	5,846 (20.5%)	4,501 (17.1%)	1,345 (59.4%)	
Sex				< 0.001
Male	13,852 (48.5%)	12,578 (47.9%)	1,274 (56.2%)	
Female	14,682 (51.5%)	13,691 (52.1%)	991 (43.8%)	
Race				< 0.001
Mexican American	4,286 (15.0%)	4,115 (15.7%)	171 (7.5%)	
Other Hispanic	2,719 (9.5%)	2,576 (9.8%)	143 (6.3%)	
Non-Hispanic White	12,634 (44.3%)	11,282 (42.9%)	1,352 (59.7%)	
Non-Hispanic Black	5,668 (19.9%)	5,209 (19.8%)	459 (20.3%)	
Other races	3,227 (11.3%)	3,087 (11.8%)	140 (6.2%)	
Education level				< 0.001
High school or below	13,042 (45.7%)	11,789 (44.9%)	1,253 (55.3%)	
College participation	8,431 (29.5%)	7,800 (29.7%)	631 (27.9%)	
College graduate or above	7,061 (24.7%)	6,680 (25.4%)	381 (16.8%)	
Smoking status				< 0.001
Yes	12,595 (44.1%)	11,190 (42.6%)	1,405 (62.0%)	
No	15,939 (55.9%)	15,079 (57.4%)	860 (38%)	
Sleep disorders				< 0.001
Yes	6,816 (23.9%)	5,941 (22.6%)	875 (38.6%)	
No	21,718 (76.1%)	20,328 (77.4%)	1,390 (61.4%)	
Hypertension				< 0.001
Yes	10,367 (36.3%)	8,688 (33.1%)	1,679 (74.1%)	
No	18,167 (63.7%)	17,581 (66.9%)	586 (25.9%)	
BMI, kg/m^2^				
Undernourished (< 18)	324 (1.1%)	295 (1.1%)	29 (1.3%)	< 0.001
Normal (18–25)	8,635 (30.3%)	8,041 (30.6%)	594 (26.2%)	
Overweight (25–30)	9,764 (34.2%)	8,952 (34.1%)	812 (35.8%)	
Obese (> 30)	9,811 (34.4%)	8,981 (34.2%)	830 (36.6%)	

Categorical variables were presented as n (percentage). CVD: cardiovascular diseases; BMI: body mass index

**Fig. 1:**
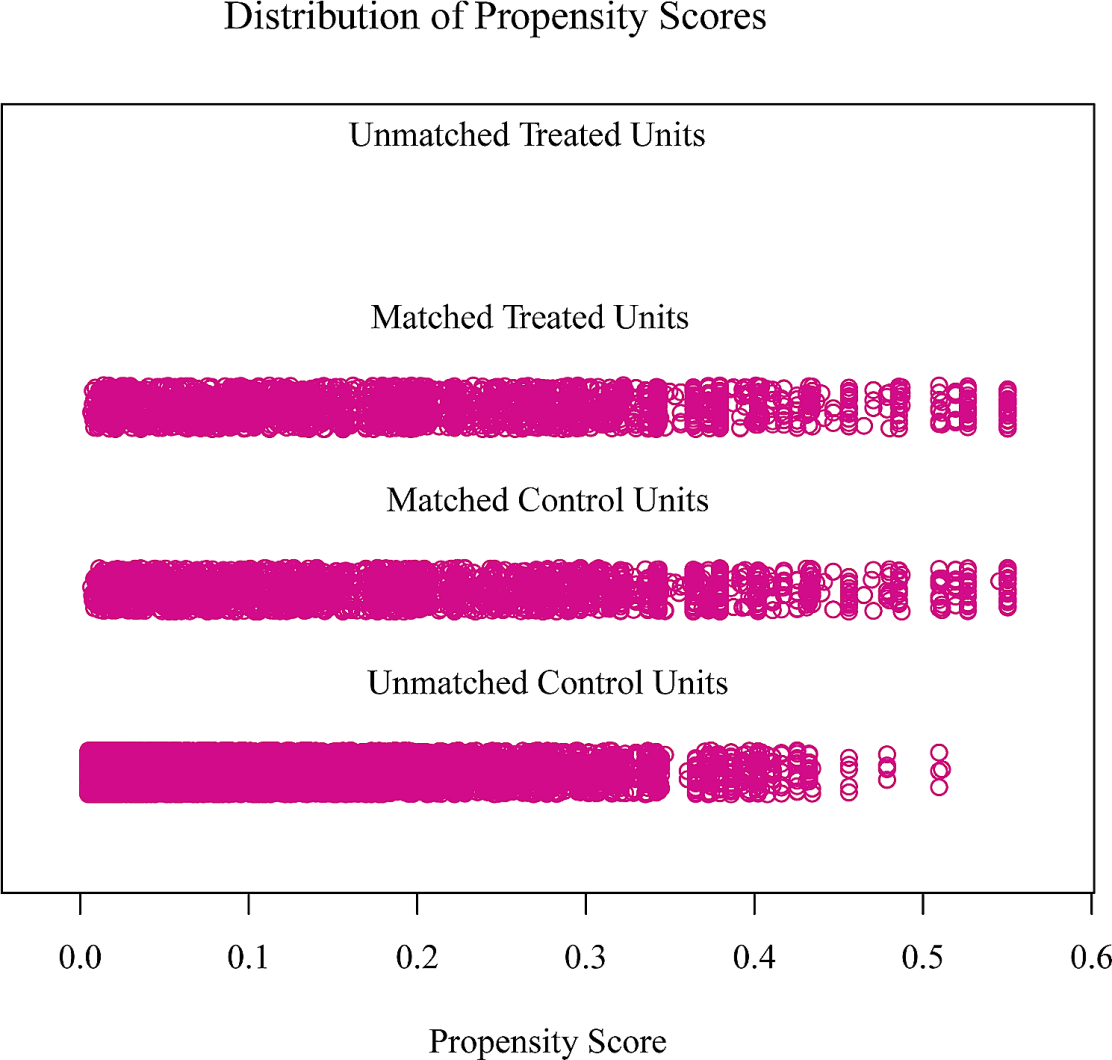
Distribution of propensity scores for the matched and unmatched treatment and control subjects with a ratio of 1:1

After PSM, 2,265 matched pairs were identified for non-CVD vs. CVD. The baseline characteristics of the two groups did not differ significantly (Table 2). Figure 1 shows the data distribution before and after matching.

**Table 2:** Characteristics of the study population after matching

Characteristic	Overall (*n* = 4,530)	Non-CVD (*n* = 2,265)	CVD (*n* = 2,265)	*P* value
Age (years)				0.963
<55years	964 (21.3%)	480 (21.2%)	484 (21.4%)	
55–<65years	867 (19.1%)	431 (19.0%)	436 (19.2%)	
≥ 65years	2,699 (59.6%)	1,354 (59.8%)	1,345 (59.4%)	
Sex				0.881
Male	2,542 (56.1%)	1,268 (56.0%)	1,274 (56.2%)	
Female	1,988 (43.9%)	997 (44.0%)	991 (43.8%)	
Race				0.925
Mexican American	348 (7.7%)	177 (7.8%)	171 (7.5%)	
Other Hispanic	275 (6.1%)	132 (5.8%)	143 (6.3%)	
Non-Hispanic White	2,713 (59.9%)	1,361 (60.1%)	1,352 (59.7%)	
Non-Hispanic Black	923 (20.4%)	464 (20.5%)	459 (20.3%)	
Other races	271 (6.0%)	131 (5.8%)	140 (6.2%)	
Education level				0.910
High school or below	2,515 (55.5%)	1,262 (55.7%)	1,253 (55.3%)	
College participation	1,249 (27.6%)	618 (27.3%)	631 (27.9%)	
College graduate or above	766 (16.9%)	385 (17.0%)	381 (16.8%)	
Smoking status				0.951
Yes	2,813 (62.1%)	1,408 (62.2%)	1,405 (62.0%)	
No	1,717 (37.9%)	857 (37.8%)	860 (38%)	
Sleep disorders				0.714
Yes	1,737 (38.3%)	862 (38.1%)	875 (38.6%)	
No	2,793 (61.7%)	1,403 (61.9%)	1,390 (61.4%)	
Hypertension				0.813
Yes	3,350 (74.0%)	1,671 (73.8%)	1,679 (74.1%)	
No	1,180 (26.0%)	594 (26.2%)	586 (25.9%)	
BMI, kg/m^2^				
Undernourished (< 18)	59 (1.3%)	30 (1.3%)	29 (1.3%)	0.999
Normal (18–25)	1,188 (26.2%)	594 (26.2%)	594 (26.2%)	
Overweight (25–30)	1,624 (35.8%)	812 (35.8%)	812 (35.8%)	
Obese (> 30)	1,659 (36.6%)	829 (36.6%)	830 (36.6%)	

Categorical variables were presented as n (percentage). CVD: cardiovascular diseases; BMI: body mass index

### Association between HbA1c levels and CVD prevalence

#### Multivariate regression model

The crude and fully adjusted correlations between HbA1c levels and CVD prevalence are presented in Table 3. The findings of the univariate logistic regression revealed a significant positive relationship between HbA1c and CVD (HbA1c Q4 vs. Q1: OR = 3.425, 95% CI = 3.021–3.882, *P* < 0.001). In the multivariate logistics regression, compared with HbA1c Q1, Q4 was associated with increased CVD prevalence (OR = 1.277, 95% CI = 1.111–1.469, *P* = 0.001). After matching in three models, a substantial link between high HbA1c levels and CVD was still seen (Table 3).

**Table 3:** Adjusted odds ratios for associations between glycated hemoglobin and the prevalence of cardiovascular disease

Model	HbA1c	Before Matching		After Matching	
		OR (95% CI)	*P* value	OR (95% CI)	*P* value
Model 1	Q1	Ref		Ref	
	Q2	1.425 (1.229–1.651)	< 0.001	1.370 (1.157–1.623)	< 0.001
	Q3	2.003 (1.760–2.280)	< 0.001	1.045 (0.879–1.242)	0.618
	Q4	3.425 (3.021–3.882)	< 0.001	1.075 (0.882–1.311)	0.472
Model 2	Q1	Ref		Ref	
	Q2	1.067 (0.914–1.245)	0.412	1.396 (1.174–1.660)	< 0.001
	Q3	1.153 (1.005–1.322)	0.043	1.059 (0.889–1.261)	0.518
	Q4	1.433 (1.251–1.641)	< 0.001	1.084 (0.888–1.323)	0.426
Model 3	Q1	Ref		Ref	
	Q2	1.053 (0.900–1.233)	0.520	1.410 (1.183–1.681)	< 0.001
	Q3	1.060 (0.921–1.219)	0.418	1.065 (0.893–1.269)	0.484
	Q4	1.277 (1.111–1.469)	0.001	1.087 (0.890–1.326)	0.414

Model 1: no covariates were adjusted

Model 2: age (<55years, 55–<65years, ≥ 65years), sex (male, female), race (non-Hispanic white, non-Hispanic black, Mexican American, other Hispanic, other races), education level (high school or below, college participation, college graduate or above) were adjusted

Model 3: age, sex, race, education level, body mass index (< 18, 18–25, 25–30, > 30), smoking status (yes, no), sleep disorders (yes, no), hypertension (yes, no) were adjusted

OR: odds ratio; CI: confidence interval. Q1:≤5.20%, Q2: 5.21–5.40%, Q3: 5.41–5.70%, Q4:>5.70%

#### Subgroup analyses

The subgroup analyses stratified by age revealed a positive association between CVD and increased HbA1c levels among people younger than 55 years (Q4 vs. Q1: OR = 2.347, 95% CI = 1.826–3.081, *P* < 0.001), which persisted up to model 3 (Q4 vs. Q1: OR = 1.437, 95% CI = 1.099–1.880, *P* = 0.008). None of the other models revealed a significant association, with the exception of the association among patients older than 65 years (Q4 vs. Q1: OR = 1.211, 95% CI = 0.992–1.478, *P* = 0.036) (Table 4).

**Table 4:** Associations between glycated hemoglobin and the prevalence of cardiovascular disease based on subgroup of age

Subgroup	HbA1c	Model1		Model2		Model3	
		OR (95% CI)	*P* value	OR (95% CI)	*P* value	OR (95% CI)	*P* value
<55years	Q1	Ref		Ref		Ref	
	Q2	1.135 (0.874–1.475)	0.343	1.102 (0.848–1.433)	0.466	0.995 (0.761–1.302)	0.973
	Q3	1.482 (1.167–1.882)	0.001	1.396 (1.098–1.775)	0.006	1.085 (0.847–1.391)	0.518
	Q4	2.347 (1.826–3.081)	< 0.001	2.111 (1.638–2.722)	< 0.001	1.437 (1.099–1.880)	0.008
55–<65 years	Q1	Ref		Ref		Ref	
	Q2	1.041 (0.730–1.484)	0.826	1.072 (0.750–1.533)	0.703	1.189 (0.825–1.714)	0.353
	Q3	1.120 (0.823–1.524)	0.472	1.117 (0.819–1.524)	0.483	1.125 (0.818–1.546)	0.469
	Q4	1.201 (0.885–1.630)	0.240	1.153 (0.848–1.569)	0.364	1.126 (0.819–1.549)	0.465
≥ 65years	Q1	Ref		Ref		Ref	
	Q2	0.938 (0.746–1.181)	0.589	0.964 (0.764–1.216)	0.758	0.978 (0.773–1.238)	0.853
	Q3	0.920 (0.751–1.125)	0.416	0.959 (0.782–1.177)	0.690	0.953 (0.775–1.173)	0.652
	Q4	1.198 (0.988–1.452)	0.066	1.232 (1.014–0.498)	0.036	1.211 (0.992–1.478)	0.060

Model 1: no covariates were adjusted

Model 2: sex (male, female), race (non-Hispanic white, non-Hispanic black, Mexican American, other Hispanic, other races), education level (high school or below, college participation, college graduate or above) were adjusted

Model 3: sex, race, education level, body mass index (< 18, 18–25, 25–30, > 30), smoking status (yes, no), sleep disorders (yes, no), hypertension (yes, no) were adjusted

OR: odds ratio; CI: confidence interval. Q1:≤5.20%, Q2: 5.21–5.40%, Q3: 5.41–5.70%, Q4:>5.70%

The subgroup analyses stratified by sex revealed a significant positive correlation between CVD and HbA1c levels among males. This correlation specifically referred to model 1 (Q4 vs. Q1: OR = 3.412, 95% CI = 2.884–4.036, *P* < 0.001) and model 3 (Q4 vs. Q1: OR = 1.346, 95% CI = 1.116–1.624, *P* = 0.002). However, the association between HbA1c and CVD was no longer present for females after removing all confounding variables from the multivariate regression model (Q4 vs. Q1: OR = 1.229, 95% CI = 0.995–1.518, *P* = 0.055) (Supplementary Table 1).

The subgroup analyses stratified by smoking status revealed a positive correlation between CVD and HbA1c levels among smokers. This correlation specifically referred to the univariate (Q4 vs. Q1: OR = 3.147, 95% CI = 2.676–3.702, *P* < 0.001) and multivariate (Q4 vs. Q1: OR = 1.422, 95% CI = 1.189–1.701, *P* < 0.001) regression models. This association was also only observed in unadjusted models for the nonsmoker group (Q4 vs. Q1: OR = 3.498, 95% CI = 2.863–4.273, *P* < 0.001) (Supplementary Table 2).

#### Restricted cubic spline

**Fig. 2:**
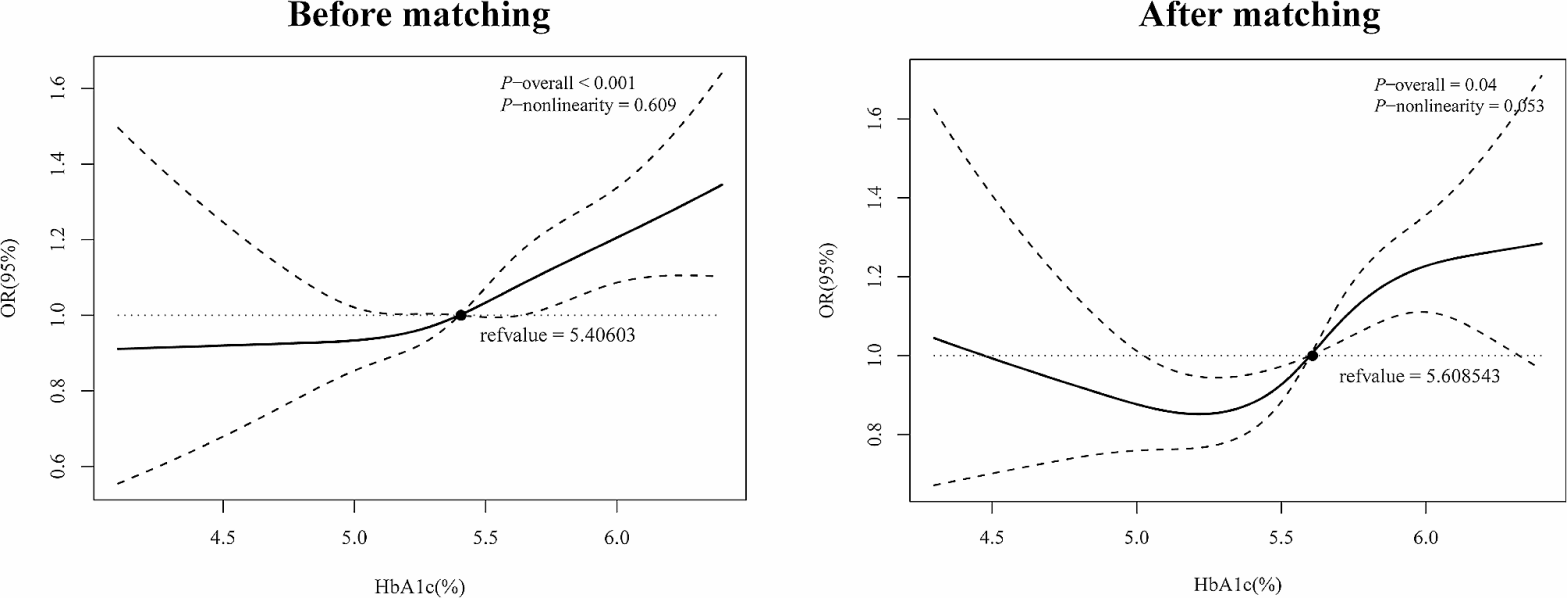
Restricted cubic spline model for the relationship between CVD risk and HbA1c levels among non-diabetics before and after matching. The 95% CIs of the adjusted ORs is between the two dotted lines. The model is adjusted for age, gender, race, education level, body mass index, smoking, sleep disorders, and hypertension. OR: odds ratio; CI: confidence interval; CVD: cardiovascular disease

After adjusting for all covariates, the RCS analysis (Fig. 2) did not reveal a nonlinear relationship between CVD and HbA1c levels (*P* for nonlinearity = 0.609 and 0.053, before and after matching, respectively).

#### Influence of the interaction between HbA1c levels and age groups on CVD

**Fig. 3:**
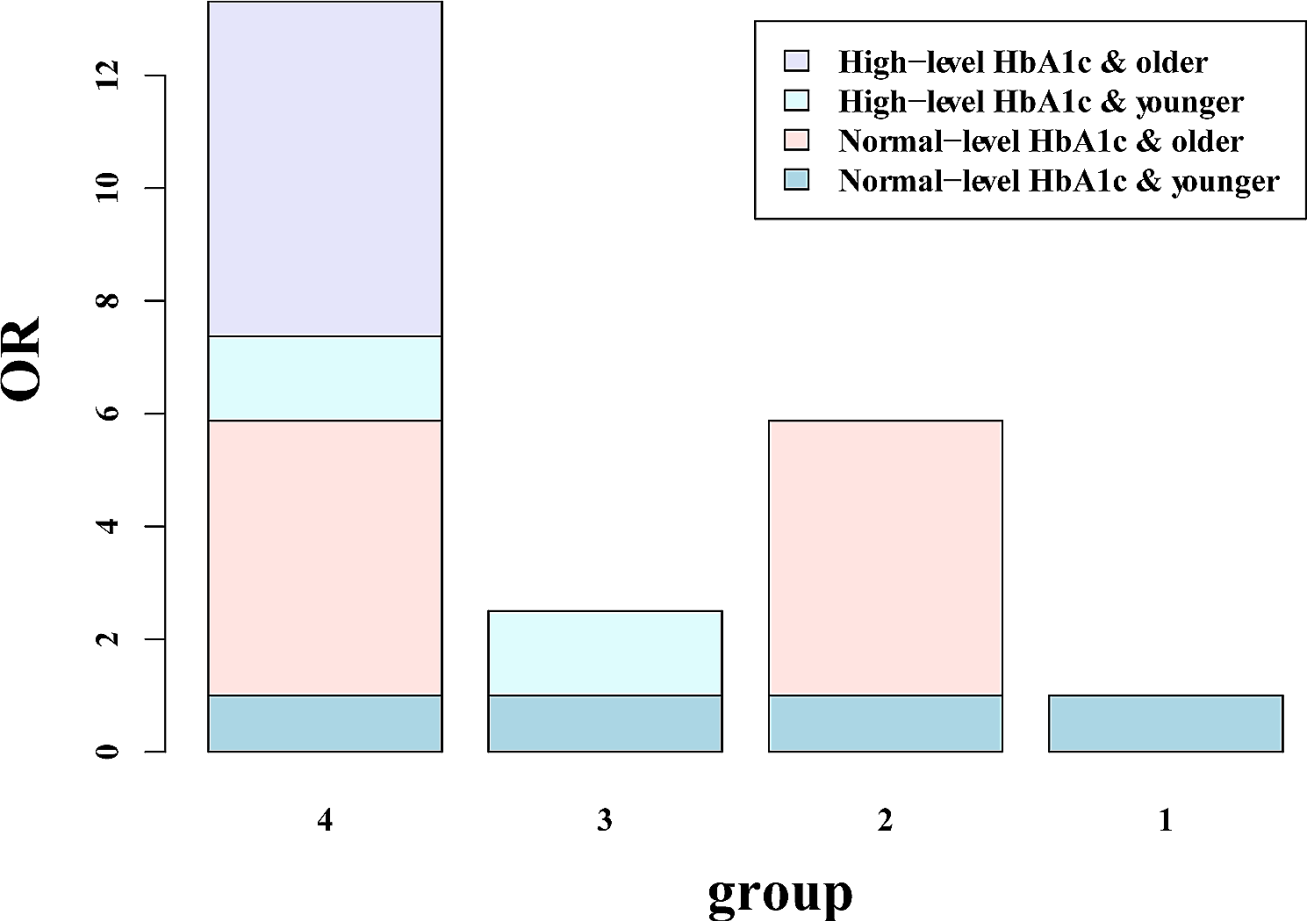
Interaction between HbA1c levels and age on CVD prevalence. The additive interaction terms of HbA1c levels and age were constructed, including normal-level HbA1c and younger people, normal-level HbA1c and older people, high-level HbA1c and younger people, high-level HbA1c and older people. The model is adjusted for gender, race, education level, body mass index, smoking, sleep disorders, and hypertension. OR: odds ratio

To establish a more in-depth model, we grouped participants with the highest HbA1c levels as high-exposure, whereas those in the first, second, and third quartiles were classified as low-exposure [15]. We also separated the population into two age groups: those aged 65 or over were categorized as the older group, while the others were classified as the younger group. In multivariate analysis, the older patients with high HbA1c levels were found to have a significantly elevated risk of CVD than those without after adjusting for all the confounders (OR = 5.941, 95% CI = 5.212–6.770). The 95% CIs of RERI and API suggested that a high HbA1c level was favorably connected with old age on CVD, with a synergistic impact. Furthermore, after correcting for all variables, the API was 0.876, indicating that the interaction between HbA1c level and age may be responsible for 87.6% of all non-diabetic CVD patients. Detailed results are shown in Table 5; Fig. 3.

**Table 5:** Logistic regression analyses of the interaction between glycated hemoglobin and age on cardiovascular disease

HbA1c	age	Model 1		Model 2		Model 3	
		OR (95% CI)	*P* value	OR (95% CI)	*P* value	OR (95% CI)	*P* value
Normal level	younger	Ref		Ref		Ref	
Normal level	older	7.527 (6.731–8.419)	< 0.001	7.400 (6.613–8.281)	< 0.001	4.874 (4.323–5.496)	< 0.001
High level	younger	2.030 (1.753–2.351)	< 0.001	1.915 (1.652–2.219)	< 0.001	1.499 (1.288–1.746)	< 0.001
High level	older	9.572 (8.470–10.817)	< 0.001	9.264 (8.190–10.480)	< 0.001	5.941 (5.212–6.770)	< 0.001
RERI (95%CI)		137.731 (95.803–179.66)		122.959 (85.181–160.737)		38.043 (25.167–50.919)	
API (95%CI)		0.942 (0.930–0.953)		0.937 (0.924–0.949)		0.876 (0.851–0.901)	
SI (95%CI)		19.224 (15.894–23.251)		9.264 (8.189–10.480)		9.698 (8.017–11.732)	

Model 1: no covariates were adjusted

Model 2: sex (male, female), race (non-Hispanic white, non-Hispanic black, Mexican American, other Hispanic, other races), education level (high school or below, college participation, college graduate or above) were adjusted

Model 3: sex, race, education level, body mass index (< 18, 18–25, 25–30, > 30), smoking status (yes, no), sleep disorders (yes, no), hypertension (yes, no) were adjusted

OR: odds ratio; CI: confidence interval: Ref: reference; RERI: relative excess risk due to interaction; AP: attributable proportion of interaction; S: synergy index. Q1:≤5.20%, Q2: 5.21–5.40%, Q3: 5.41–5.70%, Q4:>5.70%

## Discussion

This study used the NHANES 2005–2018 data set to perform an observational association analysis between HbA1c and CVD prevalence among nondiabetics. This is the first study that we are aware of to use NHANES data to demonstrate that high HbA1c levels are associated with CVD risk among nondiabetics. Moreover, this association still remained after modifying the factors that were common between HbA1c and CVD, including sex, race, age, education level, BMI, hypertension, smoking status, and sleep disorders. In the stratified analyses, the adjusted associations between HbA1c levels and CVD were significant for males, smokers, and those younger than 55 years. NHANES is distinguished by its strict sampling strategy, excellent research measurements, and thorough quality-control methods [16], which contributed to the reliability of the results obtained in our analyses.

Previous studies have found a significant association between HbA1c and CVD. However, there have been few research on the association between the two in non-diabetic patients, particularly after regulating by age. For example, a nationwide cross-sectional study found that even a 1% increase in HbA1c concentration was associated with increased mortality risks from CV or ischemic heart diseases by 40% and from all causes by about 30% [17]. At the same time, a previous Mendelian randomization revealed a detrimental effect from HbA1c on coronary artery diseases in both males and females, which was observed using a glycemic characteristic [18]. Further studies showed that HbA1c was the strongest modifiable risk factor for first and subsequent CVD events [19]. Another large national cross-sectional study found that increased HbA1c levels was strongly related to CVD risk in a community-based analysis of 11,092 nondiabetic participants [20]. With a large cohort of asymptomatic nondiabetics with low-to-moderate CV risk, a previous study also found a relationship between HbA1c levels and subclinical atherosclerosis (SA) [21]. A more comprehensive cohort study revealed that participants with prediabetes, undiagnosed diabetes, and diagnosed diabetes had greater risks of CVD compared to those with normal HbA1c [22]. These findings were consistent with the present results, showing that our findings are credible.

According to our and previous studies, the association between HbA1c and CVD may be linear. In a previous cohort study, the hypothesis of a linear relationship between HbA1c and CVD incidence rate implicit in Cox regression was verified using a penalty cubic spline [23]. A Mendelian randomization revealed no conclusive evidence of a nonlinear association between genetic correlations with major CVDs and genetic predictions of HbA1c. That study suggested that there is a linear hereditary association between HbA1c and CVDs, particularly CHD [18]. Several previous studies also included participants whose characteristics were consistent with those in our study. HbA1c > 5.4% has also been found to be linearly associated with SA in nondiabetic populations [21]. Another Mendelian randomization investigation found genetic evidence that higher mean blood glucose levels enhanced the CHD risk among nondiabetic subjects. There is no evidence that HbA1c and CHD risk had a nonlinear rather than linear relationship [24].

The mechanisms underlying the association between HbA1c levels and CVD are not understood. HbA1c is a measure of how well patients are controlling their blood sugar, and a high HbA1c level indicates prolonged hyperglycemia. A previous study suggested that HbA1c was an important type of advanced glycation end products (AGEs). AGEs can promote vasoconstriction and the migration of lymphocytes and monocytes into the vascular wall, which eventually leads to the formation of atherosclerotic plaques [25, 26]. Numerous activated immune-competent cells are present in atherosclerotic plaque, and plaque rupture causes CVD [27]. At the same time, the increased HbA1c level can also inhibit the dissociation of oxygen molecules from hemoglobin, thus aggravating myocardial ischemia and hypoxia [28]. These molecular mechanisms support the idea that elevated HbA1c levels increase the risk of CVDs.

A particularly interesting finding of the present study was that patients younger than 55 years had the strongest correlation between HbA1c and CVD prevalence. This suggests that young people with high HbA1c levels are at an increased risk of acquiring certain CVDs. Our research methodology had a cross-sectional design, which meant it was impossible to determine how the HbA1c levels of individual children and adults were affected. This means that, after stratifying by age, the impact of HbA1c levels on chronic diseases may have been underestimated. A number of studies support our position. A previous cohort study found that, when stratified by age, the correlation between HbA1c and CVD incidence rate was significant in individuals aged < 55 years, but decreased as age increased beyond that [23]. A study of 2 million Chinese adults from the Hong Kong Diabetes Surveillance Database discovered that prediabetes is related with a higher risk of severe clinical events and death, and that this relationship is stronger in younger people than in older persons [29]. Furthermore, a research that used subclinical atherosclerosis as an outcome indicator for early detection of CVD discovered that prediabetes may be less related with SA in older persons compared to middle-aged adults [30].

We found that age has an important effect on the association between HbA1c and CVD, thus we investigated the relationship further using the additive interaction model. The results showed that the elderly with high HbA1c levels had a considerably higher risk of CVD than the younger group with low HbA1c values. We found a significant synergistic association between age and HbA1c, so the stratified results must be interpreted with caution. It is important to note that even if the model produces a statistically significant interaction, this does not necessarily mean that it is biologically bound to interact [31].

Our founding demonstrated the importance of measuring HbA1c for cardiovascular risk assessment in individuals with prediabetes. The highest quartile of HbA1c levels (> 5.70%) that we used was also consistent with the American Diabetes Association’s suggested criterion for defining prediabetes in people without a diabetes diagnosis [32]. Active and effective lifestyle and drug interventions against CV risk factors in this population could effectively prevent or delay the progression of diabetes and the occurrence and development of CVDs or chronic complications from diabetes [32, 33]. More pertinent epidemiological research in Asian populations should be carried out in order to extend the importance of HbA1c measurements in the clinical practice [23].

Our investigation had several limitations. First, we were unable to identify any causal relationship because cross-sectional research measuring both exposure and outcome may lead to exposure not related to etiology [34]. Second, although we adjusted for a range of covariates, there were always some important covariates that we overlooked, such as the participants’ nutritional condition and certain comorbidities. Studies have shown that nutrition was a potential tool to regulate glucose metabolism and nutritional therapy was effective in reducing HbA1c level [35, 36]. Third, memory bias may have occurred due to the presence CVD being determined through self-reporting. Additionally, because only around 15% of individuals were matched, our matched cohort may not be typical of our whole cohort. Finally, the NHANES participants did not answer any questions about hematological illnesses, such as aplastic anemia or leukemia, and so we did not exclude people with those illnesses. It is well known that HbA1c measurements can be compromised by the presence of hemoglobinopathy, hemolytic anemia, or other conditions that affect the life span of red blood cells or interfere with the binding of glucose to hemoglobin [37]. Hematological conditions may therefore have been a confounding factor in this study.

## Conclusion

Increased HbA1c levels were associated with a high CVD incidence rate among nondiabetics. However, we still need to carefully explain the effect of age on the relationship between HbA1c and CVD in nondiabetic population. Given the correlations of HbA1c with CVDs and CV events, HbA1c might be a useful indicator for predicting CVDs and CV events in the nondiabetic population.

### Electronic supplementary material

Below is the link to the electronic supplementary material.

Supplementary Material 1

## Data Availability

All the raw data used in this study is derived from the public NHANES data portal (https://www.cdc.gov/nchs/nhanes/index.htm).
