# Supplementary material for: Age-specific differences in association of glycosylated hemoglobin levels with the prevalence of cardiovascular diseases among nondiabetics: the National Health and Nutrition Examination Survey 2005–2018
Source: BMC Cardiovasc Disord. 2024 Jun 19;24:310. doi: 10.1186/s12872-024-03978-w (PMC11186280; doi:10.1186/s12872-024-03978-w)
Supplement: Supplementary file 1 — Supplementary Material 1 [file 12872_2024_3978_MOESM1_ESM.pdf]

Age-specific Differences in Association of Glycosylated Hemoglobin Levels with the Prevalence of Cardiovascular Diseases Among Nondiabetics: the National Health and Nutrition Examination Survey 2005–2018

Contents

Supplementary Figure 1. Flow chart of the study.

Supplementary Table 1. Associations between glycated hemoglobin and the prevalence of cardiovascular disease based on subgroup of gender.

Supplementary Table 2. Associations between glycated hemoglobin and the prevalence of cardiovascular disease based on subgroup of smoking status.

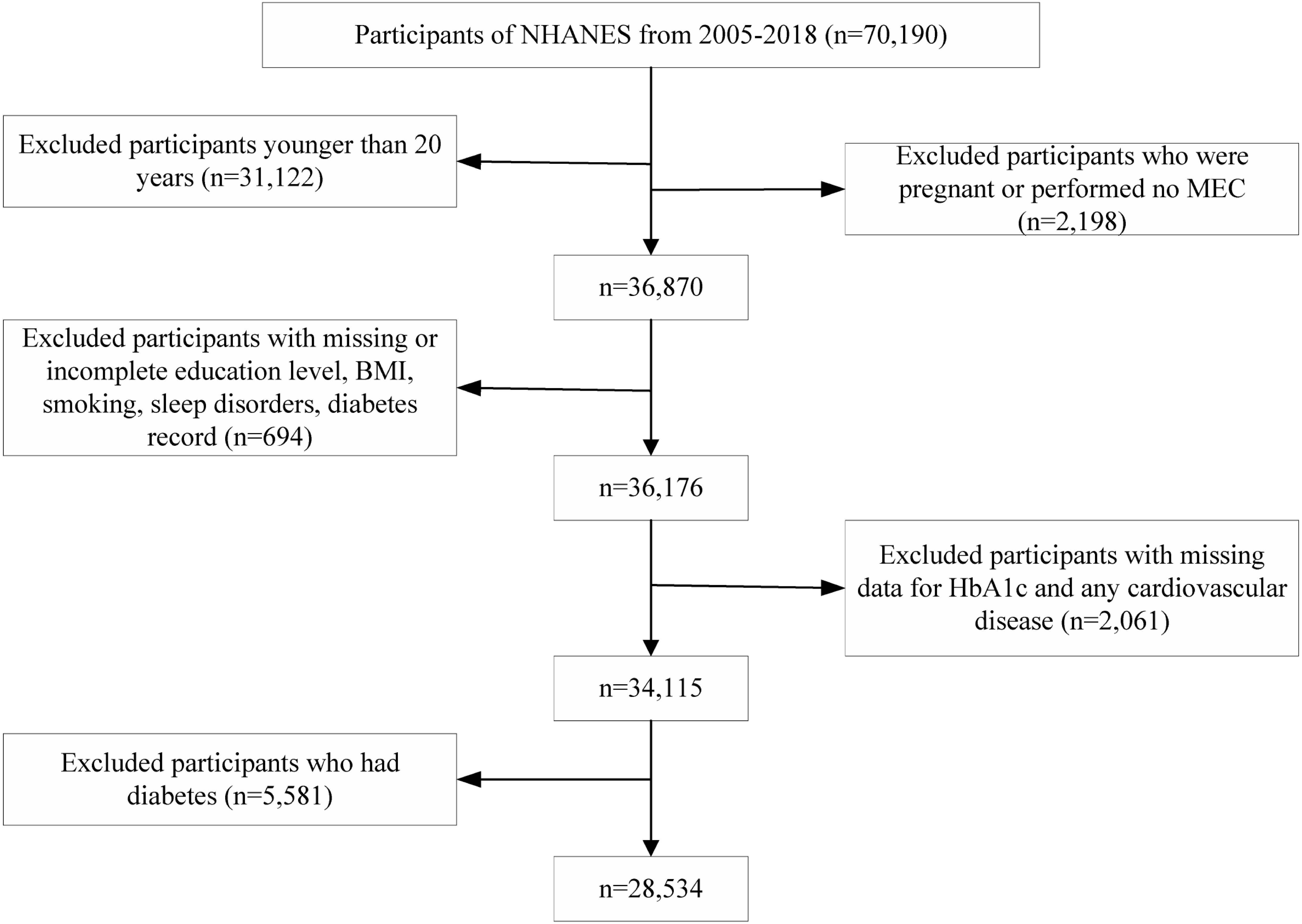
Participants of NHANES from 2005-2018 (n=70,190)
Excluded participants younger than 20 years (n=31,122)
Excluded participants who were pregnant or performed no MEC (n=2,198)
n=36,870
Excluded participants with missing or incomplete education level, BMI, smoking, sleep disorders, diabetes record (n=694)
n=36,176
Excluded participants with missing data for HbA1c and any cardiovascular disease (n=2,061)
n=34,115
Excluded participants who had diabetes (n=5,581)
n=28,534

Supplementary Table 1 Associations between glycated hemoglobin and the prevalence of cardiovascular disease based on subgroup of gender.

Subgroup	HbA1c	Model1		Model2		Model3	
		**OR (95% CI)**	***P* value**	**OR (95% CI)**	***P* value**	**OR (95% CI)**	***P* value**
Male	Q1	Ref		Ref		Ref	
	Q2	1.370(1.125–1.669)	0.002	1.046(0.849–1.290)	0.671	1.048(0.846–1.297)	0.668
	Q3	1.823(1.531–2.171)	<0.001	1.100(0.913–1.327)	0.316	1.030(0.851–1.247)	0.759
	Q4	3.412(2.884–4.036)	<0.001	1.477(1.231–1.774)	<0.001	1.346(1.116–1.624)	0.002
Female	Q1	Ref		Ref		Ref	
	Q2	1.470(1.176–1.836)	0.001	1.110(0.882–1.396)	0.375	1.067(0.844–1.350)	0.588
	Q3	2.204(1.816–2.675)	<0.001	1.253(1.022–1.536)	0.030	1.114(0.904–1.372)	0.312
	Q4	3.403(2.817–4.111)	<0.001	1.427(1.163–1.750)	0.001	1.229(0.995–1.518)	0.055

Model 1: no covariates were adjusted.

Model 2: age (＜55years, 55– < 65years, ≥65years), race (non-Hispanic white, non-Hispanic black, Mexican American, other Hispanic, other races), education level (high school or below, college participation, college graduate or above) were adjusted.

Model 3: age, sex, race, education level, body mass index (<18, 18–25, 25–30, >30), smoking status (yes, no), sleep disorders (yes, no), hypertension (yes, no) were adjusted.

OR, odds ratio; CI, confidence interval. Q1:≤5.20%, Q2: 5.21%–5.40 %, Q3: 5.41%–5.70％, Q4:>5.70%.

Supplementary Table 2 Associations between glycated hemoglobin and the prevalence of cardiovascular disease based on subgroup of smoking status.

**Subgroup**	**HbA1c**	**Model1**		**Model2**		**Model3**	
		**OR (95% CI)**	***P* value**	**OR (95% CI)**	***P* value**	**OR (95% CI)**	***P* value**
Yes	Q1	Ref		Ref		Ref	
	Q2	1.393(1.152–1.685)	0.001	1.174(0.964–1.431)	0.111	1.167(0.954–1.428)	0.133
	Q3	1.753(1.481–2.075)	<0.001	1.174(0.983–1.401)	0.076	1.086(0.906–1.301)	0.372
	Q4	3.147(2.676–3.702)	<0.001	1.596(1.342–1.899)	<0.001	1.422(1.189–1.701)	<0.001
No	Q1	Ref		Ref		Ref	
	Q2	1.398(1.103–1.773)	0.006	0.905(0.705–1.161)	0.432	0.872(0.677–1.122)	0.287
	Q3	2.229(1.816–2.734)	<0.001	1.072(0.862–1.334)	0.532	0.980(0.785–1.224)	0.858
	Q4	3.498(2.863–4.273)	<0.001	1.184(0.951–1.474)	0.132	1.058(0.846–1.323)	0.624

Model 1: no covariates were adjusted.
Model 2: age (＜55years, 55– < 65years, ≥65years), sex (male, female), race (non-Hispanic white, non-Hispanic black, Mexican American, other Hispanic, other races), education level (high school or below, college participation, college graduate or above) were adjusted.
Model 3: age, sex, race, education level, body mass index (<18, 18–25, 25–30, >30), sleep disorders (yes, no), hypertension (yes, no) were adjusted.
OR, odds ratio; CI, confidence interval. Q1:≤5.20%, Q2: 5.21%–5.40 %, Q3: 5.41%–5.70％, Q4:>5.70%.
